# The Efficacy of a Smartphone-Based App on Stress Reduction: Randomized Controlled Trial

**DOI:** 10.2196/28703

**Published:** 2022-02-15

**Authors:** Hyunchan Hwang, Sun Mi Kim, Bo Netterstrøm, Doug Hyun Han

**Affiliations:** 1 Department of Psychiatry College of Medicine Chung-Ang University Seoul Republic of Korea; 2 Department of Occupational and Environmental Medicine Bispebjerg University Hospital Copenhagen Denmark

**Keywords:** stress reduction, third-wave cognitive behavioral therapy, individual tailored treatment, randomized controlled trial, digital therapeutics

## Abstract

**Background:**

Stress management in the workplace is essential for a healthy mental and physical state. Due to technological advancements, individually tailored therapy and online cognitive behavioral therapy (CBT) are on the rise.

**Objective:**

This study analyzed the efficacy of a smartphone app based on third-wave CBT tailored to an individual.

**Methods:**

A randomized controlled trial was conducted with 126 participants who were divided into 2 groups. The intervention group used the smartphone app BetterLife for 10 weeks, while the control group was placed on a waiting list for the same duration. The Perceived Stress Scale–10 (PSS), Korean Utrecht Work Engagement Scale–9 (UWES), World Health Organization Quality of Life Assessment (WHOQOL), Beck Depression Inventory–II (BDI), and Beck Anxiety Inventory (BAI) were administered at baseline and after 10 weeks to both groups.

**Results:**

Of the 126 participants, 11 dropped out during the trial. A 2-way repeated measure analysis of covariance was conducted, controlling for baseline BDI. There were greater improvements in PSS (*F*=24.33, *P*<.001, η^2^=0.17) and UWESK scores (*F*=8.32, *P*=.0046, η^2^=0.06) in the intervention group than in the control group. WHOQOL scores exhibited statistically significant improvement in the intervention group in the overall quality of life (*F*=8.19, *P*=.0049, η^2^=0.06), physical health (*F*=8.87, *P*=.003, η^2^=0.07), psychological health (*F*=13.32, *P*<.001, η^2^=0.10), social relationships (*F*=19.43, *P*<.001, η^2^=0.14), and environmental domains (*F*=10.14, *P*=.002, η^2^=0.08) but not overall health (*F*=1.68, *P*=.20). BDI (*F*=7.17, *P*=.008, η^2^=0.06) and BAI (*F*=6.00, *P*=.02, η^2^=0.05) showed a statistically significant improvement in the intervention group, but this significance did not survive the Bonferroni correction (*P*<.005).

**Conclusions:**

These results provide evidence that smartphone-based CBT is a viable option for reducing stress in the workplace.

**Trial Registration:**

Clinical Research Information Service KCT0003231; https://cris.nih.go.kr/cris/search/detailSearch.do/15137

## Introduction

Stress management is undoubtedly crucial to the mental health as well as physical health of individuals. Chronic and high stress have been linked to depression [1], anxiety [2], coronary heart disease [3], and increased mortality [4]. Stress can be acquired from all aspects of life. Nevertheless, work-related stress has been of interest to researchers, especially in South Korea, which has one of the longest working hours within the Organization for Economic Cooperation and Development countries [5]. High stress within the workplace has also been associated with calculable economic burdens [6] and various long-term complications [7]. Therefore, stress management within the workplace is vital for both employees and employers.

Of the known methods for stress management, cognitive behavioral therapy (CBT) has been identified as effective [8,9]. Traditional CBT is performed by trained professionals such as psychiatrists or psychologists. It is usually conducted face to face or in a group setting. However, advancements in technology have allowed CBT to be computerized and administered via the internet, allowing it to be administered automatically with minimal guidance [8,10,11]. Such online CBTs have demonstrated efficacy in several areas, including the reduction of stress [12], depression, anxiety [13], and insomnia [14].

Although online CBT has advantages over traditional CBT in terms of anonymity and accessibility [15], in traditional CBT, the therapist can tailor the structured CBT to suit the client. This contrasts with most online CBTs, in which participants usually receive the same program that cannot be changed. Tailored online CBT has been implemented in treating depression and is effective, as shown in a meta-analysis [16]. However, Johansson et al [17] argued that tailored treatment is essential in addressing comorbidity, showing that tailored CBT may be more effective than ordinary internet CBT in treating depression. Stress is also associated with various disorders [1,2]. Thus, we developed an individualized CBT for stress management and performed a randomized controlled trial (RCT) to demonstrate its efficacy.

We hypothesized that using an online CBT-based app designed to help manage work stress for 10 weeks would result in a statistically significant improvement in stress-related scales compared to a waiting group.

## Methods

### Sample Size

The sample size was calculated using G*Power (Heinrich-Heine-Universität Düsseldorf), based on a statistical test on repeated measure analysis of variance (ANOVA), since the covariance number was not determined. A type I error of .05 and statistical power of 0.8 were used. The correlation among repeated measures was set conservatively at 0, and based on previous similar studies [8], a mild to moderate effect size was estimated (effect size *f*=0.20). Based on these calculations, 51 participants were needed in each group, and after calculating a 20% dropout rate, the required total sample size was set at 128 participants.

### Participants

Participants were recruited through billboard advertisements between November 2019 and January 2020. The inclusion criteria for the trial were as follows: (1) elevated perceived stress defined by a score of 14 or higher on the Perceived Stress Scale–10 (PSS); (2) indication that the reason for stress was mainly work-related (the reason for stress was considered to be work-related if the participant could identify one or more stress-related factors that they experienced in the work environment and stress in other areas such as family were minor in comparison to work-related stress); (3) employment of at least 20 hours per week and not self-employed; (4) aged 18 to 60 years; and (5) ability to provide informed consent to participate after being given information about the trial and other information that the participant must know to participate. The exclusion criteria were as follows: (1) inability to provide informed consent; (2) education level below the 9th grade; (3) history of congenital brain disorder, cerebral palsy, or other acquired brain injuries; (4) history of neurological disorders; (5) severe anxiety, depression, or psychotic disorder as assessed by the Korean Symptom Checklist–95; or (6) history of drug or alcohol abuse.

**Figure 1:**
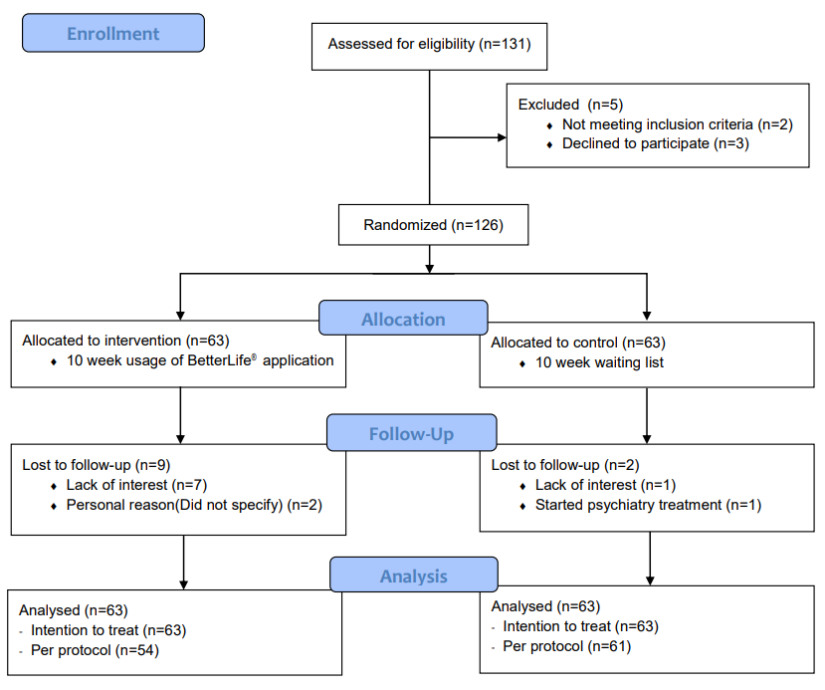
CONSORT 2010 flow diagram of enrollment.

In total, 131 individuals who had work-related stress expressed interest in the trial. Of these, 3 withdrew participation after being informed of the detailed trial protocol; written consent was received from the remaining 128 participants. Two patients were excluded because of clinically relevant scores in the Korean Symptom Checklist–95. A total of 126 participants were included in the trial and randomized into 2 parallel groups with equal allocation ratios (Figure 1). Randomization was performed by an external researcher from a separate institute, and simple randomization via a random number generator using Excel (Microsoft Corp) was used. Each participant was assigned a random trial number at enrollment, and the external researcher was only provided with the individual trial number to ensure blinded randomization. The researcher performing data analysis (DHH) was also provided only with the individual trial number to blind the outcome assessment.

The intervention group was given a random individual application ID to access the app, a link for downloading the app, and a manual of the program. Further, they were instructed to use the BetterLife program for 50 minutes per week for 10 weeks. They had the option to contact a designated person to ask for help in operating the app. Participants were sent a text message as a prompt to notify them of their app use every week during the trial. Members of the control group were placed on a waiting list for 10 weeks after which they were given access to the BetterLife program for the next 10 weeks. This was primarily done for ethical reasons, and data collected from the control group after using the BetterLife program were not used in this analysis. These data are being considered for use in secondary analyses, which can strengthen our research. This trial was approved by the institutional review board of Chung-Ang University Hospital (1712-008-304) and registered in the Clinical Research Information Service of Republic of Korea (KCT0003231), a member of the WHO International Clinical Trials Registry Platform. All participants enrolled in the trial provided written informed consent.

Of the 126 participants, 11 dropped out of the trial: 9 from the intervention group (14.3%) and 2 from the control group (3.2%). Of the 9 participants who were excluded from the intervention group, 2 left the trial due to personal reasons and 7 wished to leave due to lack of interest. Of the 2 participants who were excluded from the control group, 1 wished to leave the trial due to lack of interest and 1 was excluded because they were starting psychiatric treatment. Finally, 115 participants—54 in the intervention group and 61 in the control group—completed the 10-week trial and post-10-week data were collected. The 11 excluded participants’ post-10-week data were produced by the baseline observation carried forward method, and data were analyzed using an intention-to-treat method. The average use time of the program was 56.40 (SD 8.42) minutes per week; this was calculated by using the program time of the 54 participants who finished the 10-week program only. No substantial harm or unintended effects were observed.

### Data Collection

Participant demographic data were collected including age, sex, education level, marital status, alcohol consumption, and smoking habits. Information on employment was also collected, including company size, work field, type of employment, job grade, whether they handle direct customer complaints, income, workdays per week, work hours per day, work experience in the current work field, and job. Information regarding the number of late days, early leave days, and absent days of the past month was also collected. The participants were asked to complete the PSS, Korean Utrecht Work Engagement Scale–9 (UWES), abbreviated version of the World Health Organization Quality of Life Assessment (WHOQOL), Beck Depression Inventory–II (BDI), and Beck Anxiety Inventory (BAI) to assess psychological and work stress-related status at baseline and after the 10-week intervention. PSS and the overall quality of life score of the WHOQOL at 10 weeks were set as the primary outcomes. Data were collected in a face-to-face setting by the researchers of Chung-Ang University Hospital at a separate location preferred by the participant. Participants received 100,000 Won (US $83) as compensation for their travel fees.

#### Perceived Stress Scale–10

The PSS was developed by Cohen et al [18] and validated in Korea by Park et al [19]. It is a widely used scale consisting of 10 items on a 5-point scale and is designed to measure the degree of stress experienced by individuals, with a high score indicating high perceived stress. Although the scale authors did not develop the scale with a cutoff value, the scores are often divided into 3 parts for clinical use: 0 to 13, low stress; 14 to 26, moderate stress; and >27, severe stress [20].

#### Korean Utrecht Work Engagement Scale–9

UWES is one of the most frequently used scales related to work engagement. It calculates work engagement by measuring vigor, dedication, and work absorption through a 7-point frequency rating scale ranging from 0 (never) to 6 (always). It was developed by Schaufeli et al [21] and has been validated in Korea by Kim et al [22].

#### World Health Organization Quality of Life Assessment, Abbreviated

WHOQOL was developed by the WHO as a measure to assess the quality of life (QoL) in individuals across different cultures [23]. It has been validated in Korea by Min et al [24]. The WHOQOL consists of 26 questions and uses a 5-point scale from 1 to 5, with a higher score indicating better QoL. One question pertains to overall perception of QoL (overall QoL); another pertains to overall perception of health (overall health). The remaining 24 questions were calculated to measure an individual’s QoL in each of 4 domains: physical health, psychological health, social relationships, and environment. The raw scores were converted into transformed scores (0 to 100) using the developers’ suggested method for easier comparison.

#### Beck Depression Inventory–II

The BDI was initially developed by Beck in 1961 and revised in 1979 to BDI-IA and finally to BDI-II in 1996 to accommodate the changes in the *Diagnostic and Statistical Manual of Mental Disorders, fourth edition* diagnosis for depressive disorders [25]. It is a self-report questionnaire with 21 items, and each item is rated on a 4-point scale from 0 to 3. It has been validated in many countries across the world, including Korea [26]. The BDI was designed to measure the depressive symptoms of an individual during the past 2 weeks.

#### Beck Anxiety Inventory

The BAI was developed by Beck in 1988 [27]. It is a self-report questionnaire with 21 items. Similar to the previous BDI, it rates each item from 0 to 3 on a 4-point scale. The BAI is designed to measure anxiety independent of depressive symptoms and was validated in Korea by Yook et al [28] in 1997.

### Intervention

BetterLife is a smartphone-based program for the treatment of stress, depression, anxiety, and sleep disorders used for guided self-help therapy; it can be used for both prevention and treatment. It is designed for anonymous treatment, targeting individuals with mild or moderate symptoms. BetterLife uses recognized manuals for CBT and problem-solving therapy and consists of approximately 600 modules of 7 types: test modules, psychoeducational modules, cognitive exercises, practical exercises, diary modules, notification modules, and comment modules. The test modules included the World Health Organization Well-Being Index (WHO-5) [29], the Major Depression Inventory (MDI) [30,31], the General Anxiety Test–7 (GAD-7) [32], and a short version of the Copenhagen Psychosocial Questionnaire [33]; the latter is used for assessment of work-related stressors. Psychoeducational modules are e-learning modules that provide knowledge about illnesses, treatment methods, and background information. They have talking-head videos with an overlay of animated graphical visuals. Cognitive exercises are interactive exercises programmed in HTML5, providing opportunities for users to work with their thoughts and feelings. The interactivity makes it possible for the user to work with their data registered in a database on a server. Practical exercises include physical exercises, relaxation exercises such as meditation, and exercises away from home. Diary modules are used to map activities and the development of mental conditions over time. Data from the registrations in the diary modules are saved so the user can see them. Notification modules ensure the user’s adherence to the treatment flows, and comment modules provide feedback to the user’s treatment progression. Comment modules follow tests and exercises.

All treatment components in the program are based on evidence-based methods documented in scientific studies [11,34]. BetterLife is powered by an advanced treatment flow engine that enables individualized transdiagnostic treatment and continuous follow-up of treatment results carried out in a dialog form with the user. This dialog also includes an online dialog with an attached therapist and a chosen friend (helper) who can help the user understand the psychoeducational parts and conduct cognitive and practical exercises. The tests in the program are used both for reference to the individual treatment interventions in the program and for the continuous monitoring of treatment progression. If severe symptoms are detected, the user is referred for external psychological help in a clinic or hospital—in case of suicidal thoughts by call function directly to an acute clinic. The user sees graphical charts of the test result history to experience treatment progression. BetterLife has a toolbox with all tests and exercises in the program from where the user can choose to redo a test or exercise at any time. The primary test and exercise results are always visible on the main page of the BetterLife program.

The chance to use the WHO-5 questionnaire was provided every week to tailor to each user. Thus, the user could follow the improvement over time, even in a graph. If the test value is low (meaning low well-being), the user is referred to the depression (MDI) and anxiety (GAD-7) modules. When acceptable values of these tests are obtained (showing a low degree of depression and anxiety symptoms), the user automatically returns to the other parts of the program. Another feature to personalize the program to the user is the user’s own estimation of stressors in work and daily life. The user is guided to modules specifically concerning the problems pinpointed by the user. An overview of the program can be found in the supplements (Multimedia Appendices 1 and 2).

### Statistical Analysis

All data were analyzed using SPSS (version 19.0, IBM Corp). The data presented here concern the intervention group compared to the waiting list group (controls) at baseline and follow-up. Data on the controls after using the BetterLife program and those on the program’s long-term effects are not presented. Baseline information, including demographic and psychological scales, was compared using the chi-square test, Fisher exact test, and independent *t* test. Intervention outcomes were measured using work-related scales and psychological scores. Changes in late days, early leave days, and absent days in the past month were also collected to determine whether the app could have a tangible effect on work life.

For all participant data, univariate logistic regression analyses were conducted to determine whether variables of independent factors—such as age, gender ratio, education level, marital status, alcohol consumption, smoking, BDI scores, BAI scores, type of work field, workdays, work hours, and work experience—could explain a significant amount of variance in the dependent variable (high perceived stress) after considering all other variables. Statistically significant independent factors were evaluated using a multivariate logistic regression analysis using a stepwise forward conditional method. High perceived stress was defined as a PSS score of 27 or higher.

Using 2-way repeated-measures analysis of covariance (ANCOVA; split-plot ANCOVA) with time as a within-factor and group as a between-factor, controlling covariates determined from the logistic regression and the changes in PSS, UWES, WHOQOL, BDI, and BAI scores were calculated. Bonferroni correction was used to compensate for multiple comparisons, and α=.05/10=.005. *P*<.005 was considered statistically significant. The number of late days, early leave days, and absent days were analyzed separately by 2-way repeated measure ANOVA with time as a within-factor and group as a between-factor since they were not collected as part of the initial intervention outcome.

## Results

### Comparison of Demographic Characteristics and Psychological Scale Scores between the Intervention and Control Groups

There were no significant differences in age, gender ratio, education level, marital status, alcohol consumption, smoking, company size, work field, type of employment, job grade, direct customer complaint handling, workdays, work hours, work experience, and late days, early leave days, and absent days in the past month between the intervention and control groups (Table 1). At baseline, there were no significant differences in PSS, UWES, WHOQOL, BDI, and BAI scores (Table 2).

**Table 1:** Comparison of demographic characteristics of participants between intervention and control groups.

				Intervention (n=63)		Control (n=63)		Test statistic		*P* value
**Demographic information**										
	Age (years), mean (SD)		38.6 (9.8)		37.3 (8.8)		0.75^a^		.46	
	**Gender, n (%)**		—^b^		—		2.45^c^		.12	
		Male	9 (14)		16 (25)		—		—	
		Female	54 (86)		47 (75)		—		—	
	**Education, n (%)**		—		—		0.89^c^		.64	
		High school	12 (19)		10 (16)		—		—	
		Undergraduate	46 (73)		45 (71)		—		—	
		Graduate	5 (8)		8 (13)		—		—	
	**Marital state, n (%)**		—		—		1.56^c^		.21	
		Single	26 (41)		33 (52)		—		—	
		Married	37 (59)		30 (48)		—		—	
	**Alcohol, n (%)**		—		—		0.05^c^		.82	
		Yes	51 (81)		52 (83)		—		—	
		No	12 (19)		11 (18)		—		—	
	**Smoking, n (%)**		—		—		2.49^c^		.12	
		Yes	3 (5)		8 (13)		—		—	
		No	60 (95)		55 (87)		—		—	
**Workplace information**										
	**Company size (employee), n (%)**		—		—		2.24^d^		.72	
		<10	5 (8)		5 (8)		—		—	
		10-29	2 (3)		6 (10)		—		—	
		30-99	11 (18)		11 (18)		—		—	
		100-299	5 (8)		4 (6)		—		—	
		≥300	40 (64)		37 (59)		—		—	
	**Type of work field, n (%)**		—		—		2.23^d^		.85	
		Sales and services	3 (5)		1 (2)		—		—	
		Technical	2 (3)		3 (5)		—		—	
		Office	16 (25)		21 (33)		—		—	
		Professional	23 (37)		22 (35)		—		—	
		Civil servant/teacher	5 (8)		4 (6)		—		—	
		Other^e^	14 (22)		12 (19)		—		—	
	**Type of employment, n (%)**		—		—		0.95^c^		.33	
		Regular position	51 (81)		55 (87)		—		—	
		Temporary position	12 (19)		8 (13)		—		—	
	**Job grade, n (%)**		—		—		3.27^c^		.66	
		Staff	2 (3)		1 (2)		—		—	
		Administrative manager	12 (19)		12 (19)		—		—	
		Assistant manager	10 (16)		14 (22)		—		—	
		General manager	12 (19)		17 (27)		—		—	
		Director and higher	18 (29)		13 (21)		—		—	
		No job grade	9 (14)		6 (10)		—		—	
	**Customer complaints, n (%)**		—		—		0.93^c^		.34	
		Yes	41 (65)		46 (73)		—		—	
		No	22 (35)		17 (27)		—		—	
	**Income (KRW^f^), n (%)**		—		—		3.23^d^		.53	
		<₩2 million	15 (24)		10 (16)		—		—	
		₩2-3 million	30 (48)		34 (54)		—		—	
		₩3-4 million	8 (13)		9 (14)		—		—	
		₩4-5 million	5 (8)		8 (13)		—		—	
		>₩5 million	5 (8)		2 (3)		—		—	
	Work days/week^g^, mean (SD)		5.0 (0.3)		5.1 (0.2)		–0.72^a^		.47	
	Work hours/day^g^, mean (SD)		8.2 (0.9)		8.3 (0.7)		–1.16^a^		.25	
	Work experience in current work field (months)^g^, mean (SD)		133.2 (103.6)		128.0 (91.8)		0.30^a^		.77	
	Work experience in current job (months)^h^, mean (SD)		107.3 (103.4)		91.2 (90.7)		0.92^a^		.36	
	Number of late days in past month, mean (SD)		0.7 (1.8)		0.8 (1.9)		–0.29^a^		.77	
	Number of early leave days in past month, mean (SD)		0.3 (0.6)		0.3 (0.9)		–0.34^a^		.73	
	Number of absent days in past month, mean (SD)		0.1 (0.2)		<0.1 (0.1)		1.01^a^		.31	

^a^2-tailed *t* test.

^b^Not applicable.

^c^Chi-square test.

^d^Fisher exact test.

^e^Other includes miscellaneous positions in the company <9 employees, company management position >10 employees, etc.

^f^KRW: South Korean Won. 1198 Won=$1 USD.

^g^One value missing.

^h^Four values missing.

**Table 2:** Comparison of baseline work stress-related scale scores.

			Intervention (n=63)		Control (n=63)		Test statistic^a^		*P* value
PSS^b^			21.6 (5.9)		20.1 (3.8)		1.67		.10
UWES^c^			2.6 (0.8)		2.8 (0.8)		–1.41		.16
**WHOQOL^d^**									
	Overall QoL^e^	3.0 (0.8)		3.1 (0.7)		–1.15		.25	
	Overall health	2.9 (0.9)		2.9 (0.8)		0.21		.84	
	Physical health	56.1 (13.8)		58.4 (12.3)		–0.99		.33	
	Psychological	52.5 (15.1)		57.3 (12.9)		–1.92		.06	
	Social relationship	55.1 (18.1)		61.0 (15.8)		–1.94		.06	
	Environmental	58.2 (14.6)		60.4 (12.6)		–0.88		.38	
BDI^f^			17.7 (9.5)		15.3 (7.7)		1.53		.13
BAI^g^			13.8 (9.3)		11.0 (7.3)		1.89		.06

^a^2-tailed *t* test.

^b^PSS: Perceived Stress Scale.

^c^UWES: Utrecht Work Engagement Scale.

^d^WHOQOL: World Health Organization Quality of Life Scale.

^e^QoL: quality of life.

^f^BDI: Beck Depression Inventory.

^g^BAI: Beck Anxiety Inventory.

### Comparison of Symptom Improvement Between the Intervention and Control Groups

In logistic regression analysis, only baseline BDI scores in all participants were positively correlated with high perceived stress (B=0.20, Exp(B)=1.22, *P*<.001; Table 3). A separate paired *t* test of the repeated measures of the control group showed no significant differences after the Bonferroni correction (Multimedia Appendix 3).

**Table 3:** Results of univariate and multivariate logistic regression analysis for high perceived stress.^a^

		B^b^	*P* value	Exp(B)^c^
**Univariate**				
	Age	0.03	.91	1.00
	Gender	–0.22	.72	0.80
	Education	—^d^	.68	—
	Marital state	–0.28	.59	0.76
	Alcohol	0.34	.59	1.40
	Smoking	0.46	.67	1.58
	Company size	—	.89	—
	Types of work field	—	.87	—
	Type of employment	0.98	.10	2.67
	Job grade	—	.86	—
	Customer complaints	–0.06	.92	0.94
	Income	—	.61	—
	Work days/week	–21.03	>.99	0
	Work hours/day	–0.40	.27	0.67
	Work experience in current work field	<0.01	.53	1.00
	Work experience in current job	<0.01	.61	1.00
	BDI^e^	0.20	<.001	1.22
	BAI^f^	0.14	<.001	1.15
**Multivariate**				
	BDI	0.20	<.001	1.22

^a^Multivariate logistic regression analysis was conducted with BDI and BAI as independent variables.

^b^B: logistic regression coefficient.

^c^Exp(B): e to the power of B (odds ratio).

^d^Not applicable.

^e^BDI: Beck Depression Inventory–II.

^f^BAI: Beck Anxiety Inventory.

**Figure 2:**
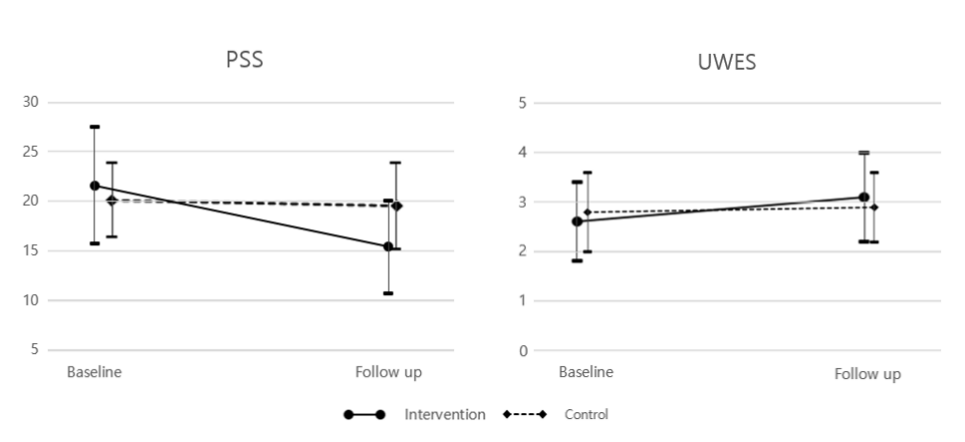
Repeated measure analysis of covariance controlling baseline Beck depressive inventory scores. Left: comparison of changes in Perceived Stress Scale (PSS) scores between intervention group and control group (*F*=24.33, *P*<.001). Right: comparison of changes in the Utrecht Work Engagement Scale–Korean Version (UWES) scores between intervention group and control group (*F*=8.32, *P*=.0046).

In the split-plot ANCOVA, there was homogeneity of variances (*P*>.05) and covariances (*P*>.001), as assessed by the Levene test of homogeneity of variances and Box M test, respectively, in all categories except for the number of late days, early leave days, and absent days in the past month. The Mauchly test of sphericity was ignored because there were only two groups for each factor. Controlling baseline BDI scores, the intervention group showed greater improvement in the changes in PSS (*F*=24.33, *P*<.001, η^2^=0.17) and UWESK scores (*F*=8.32, *P*=.0046, η^2^=0.06) compared to the control group (Figure 2). WHOQOL scores also demonstrated a statistically significant interaction between treatment groups and pretests/posttests in overall QoL (*F*=8.19, *P*=.0049, η^2^=0.06) and physical health (*F*=8.87, *P*=.003, η^2^=0.07), psychological (*F*=13.32, *P*<.001, η^2^=0.10), social relationships (*F*=19.43, *P*<.001, η^2^=0.14), and environmental domains (*F*=10.14, *P*=.002, η^2^=0.08) but not overall health (*F*=1.68, *P*=.20; Table 4). Although BDI (*F*=7.17, *P*=.008) and BAI (*F*=6.00, *P*=.02) showed traditionally low *P* values, they did not survive the Bonferroni correction. Unadjusted ANOVA yielded similar results except for BDI (*F*=9.67, *P*=.002, η^2^=0.07), which showed a significant interaction between treatment groups and pretests/posttests (Multimedia Appendix 4).

**Table 4:** Results of split-plot analysis of covariance for stress-related factors.

			Intervention (n=63)					Control (n=63)					Test statistics^a^					*P* value^b^
			Baseline		Follow-up		Baseline			Follow-up		*F* test^c^			η^2^			
PSS^d^			21.6 (5.9)		15.4 (4.7)		20.1 (3.8)			19.6 (4.4)		24.33			0.17		<.001	
UWESK^e^ total			2.6 (0.8)		3.1 (0.9)		2.8 (0.8)			2.9 (0.7)		8.32			0.06		.0046	
**WHOQOL^f^**																		
	Overall QoL^g^	3.0 (0.8)		3.6 (0.7)		3.1 (0.7)			3.3 (0.8)		8.19			0.06		.0049		
	Overall health	2.9 (0.9)		3.3 (0.8)		2.9 (0.8)			3.0 (0.9)		1.68			—^h^		.20		
	Physical health	56.1 (13.8)		65.8 (14.1)		58.4 (12.3)			60.7 (11.8)		8.87			0.07		.003		
	Psychological	52.5 (15.1)		63.6 (16.7)		57.3 (12.9)			59.5 (12.3)		13.32			0.10		<.001		
	Social relationship	55.1 (18.1)		66.6 (14.6)		61.0 (15.8)			59.1 (16.3)		19.43			0.14		<.001		
	Environmental	58.2 (14.6)		68.6 (12.5)		60.4 (12.6)			61.9 (10.9)		10.14			0.08		.002		
BDI^i^			17.7 (9.5)		11.5 (9.2)		15.3 (7.7)			13.1 (7.6)		7.17			0.06		.008	
BAI^j^			13.8 (9.3)		8.0 (8.7)		11.0 (7.3)			9.0 (7.0)		6.00			0.05		.02	

^a^Statistics reported are for the interaction between intervention and time of each variable.

^b^*P*<.005 is considered significant.

^c^degree of freedom: 1123.

^d^PSS: Perceived Stress Scale.

^e^UWESK: Utrecht Work Engagement Scale.

^f^WHOQOL: World Health Organization Quality of Life Scale.

^g^QoL: quality of life.

^h^Not applicable.

^i^BDI: Beck Depression Inventory–II.

^j^BAI: Beck Anxiety Inventory.

Similarly, in the repeated measures ANOVA, homogeneity of variances (*P*>.05) and covariances (*P*>.001) were assessed by the Levene test of homogeneity of variances and Box M test, and the Mauchly test of sphericity was ignored. There were no significant interactions in the number of late days, early leave days, and absent days in the past month between the treatment groups and pretests/posttests (Table 5).

**Table 5:** Results of split-plot analysis of variance for the additional outcome.

	Intervention (n=63)		Control (n=63)		Test statistic^a,b^	*P* value
	Baseline	Follow-up	Baseline	Follow-up		
Number of late days in past month	0.7 (1.8)	0.4 (1.1)	0.8 (1.9)	0.7 (1.7)	0.48	.49
Number of early leave days in past month	0.3 (0.6)	0.1 (0.4)	0.3 (0.9)	0.3 (0.9)	0.74	.39
Number of absent days in past month	0.1 (0.2)	<0.1 (0.2)	<0.1 (0.1)	<0.1 (0.3)	0.49	.48

^a^Statistics (*F* test) reported are for the interaction between intervention and time of each variable.

^b^degree of freedom: 1124.

### Sensitivity and Dropout Analyses

A sensitivity analysis was performed with the 54 intervention group and 61 control group participants who completed the trial. These data were evaluated separately using per-protocol analysis. Results showed a significant interaction between treatment groups and pretests/posttests in all scales except for the overall health of WHOQOL. Compared to the intention-to-treat analysis, the per-protocol analysis showed overall more significant (lower) *P* values and higher effect sizes (Multimedia Appendix 5). Little difference was found between the 2 analyses on the number of late days, early leave days, and absent days (Multimedia Appendix 6).

Dropout analysis was also performed, comparing the baseline values of the 11 participants who dropped out and the 115 participants who completed the trial. In the demographic data, work hours per day were significantly lower in the dropout group (*t*=3.63, *P*<.001; Multimedia Appendix 7). When comparing the baseline work-related stress and psychological scales, the dropout group showed significantly lower PSS scores (*t*=3.63, *P*<.001) and social relationship scores in the WHOQOL (*t*=–2.19, *P*=.03; Multimedia Appendix 8). The number of early leave days was also significantly lower in the dropout group (*t*=4.25, *P*<.001).

## Discussion

### Principal Findings

The BetterLife app, a program developed for the management of stress, effectively improved the degree of stress in people with work-related stress measured by PSS compared with control groups on a waiting list. It also improved work engagement as determined by the UWES. Furthermore, QoL improved in all domains except for overall health, according to the WHOQOL. However, the total number of late days, early leave days, and absent days showed no improvement in the 2 groups.

### Intervention Effectiveness on Stress Reduction and Work Engagement

The effects of CBT on stress management in online settings have been well documented in previous studies [8,35]. A meta-analysis of web- and computer-based stress management interventions showed that these interventions effectively reduced stress and, on average, had a moderate effect size (Cohen *d*=0.43) on stress reduction [8]. However, recent studies have shown larger effect sizes. Asplund et al [36] identified a significant reduction in stress with a moderate to large effect size through an RCT with a guided internet-based stress management intervention. Ebert et al [10] also observed a large effect size in stress reduction using an internet- and mobile-based stress management program [10]. Our study showed that the intervention group had a significantly higher reduction in the PSS score from baseline to posttreatment when compared to the control group with a large effect size (*F*=24.83, *P*<.001, η^2^=0.17). We estimate this large effect size to be because of meditation, emotional acceptance, and other third-wave CBT qualities incorporated in the program. Asplund et al [36] and Ebert et al [10] also used parts of third-wave CBT interventions, which could explain the large effect sizes of the results. Third-wave CBT helps participants to be more aware and accepting of their thoughts through ways such as meditation or behavior activation [37]. It has demonstrated strong efficacy in stress reduction [38].

The intervention group also showed improved work engagement, represented by improvements in the UWES. Work engagement, often considered the opposite of burnout, is a positive work-associated mindset with characteristics such as vigor, dedication, and absorption [39]. Extensive literature supports the positive benefits of work engagement. Halbesleben et al [40] demonstrated, through a study conducted on 587 employees in various occupations, that work engagement shared variance with job performance, implying that developing work engagement leads to positive outcomes in job performance and job retention. A meta-analysis of 7939 business units concluded that work engagement is related to meaningful business outcomes [41]. The authors of this study also implied that this improvement in employee work engagement and satisfaction could increase profits for businesses. Work engagement has also been known to affect absenteeism [42] and overall sickness absence [43]. However, our data did not show a significant difference in the number of sick days, early leave days, and absent days between baseline and postintervention. We estimate this to be because of the small number of sick, early leave, and absent days at baseline, subsequently requiring more statistical power than the trial’s original design. However, several similar studies failed to demonstrate a significant improvement in sick leave days even with symptom improvement [44], which could imply that symptom improvement through CBT alone might not be enough for a tangible improvement in sick leave.

### Intervention Effectiveness on Quality of Life

Improvement of QoL in all domains, including overall QoL, was seen in the intervention group. However, the perception of overall health did not differ between the control and intervention groups. QoL improvement by CBT has been observed in literature: Hofmann et al [45] used a meta-analysis to observe the effect of CBT on QoL in anxiety disorders and found it to be effective. An RCT conducted using an internet-based self-guided stress management intervention for employees, similar to that in this study, also exhibited improvements in QoL [10], but only the mental health component of QoL improved (not the physical health component), which was similar to our findings. Although the physical domain of WHOQOL in our trial showed significant improvements, the effect size was the smallest among the 4 domains, and overall perception of health showed no significant improvement. This may be because improving physical health and related QoL is significantly more complex than improving mental health and related QoL, although possible. Therapies with a meditative component, such as yoga and mindfulness-based cognitive therapy, have been shown to improve health-related QoL [46]. CBT that improves sleep disturbance also induces an improvement in health-related QoL [47]. Furthermore, our program had separate modules dedicated to sleep improvement and meditation. A few participants gave positive feedback on the meditation module, which could have resulted in the mixed health-related QoL improvements. Future programs should emphasize this in CBT for further improvement in physical health.

The social relationship domain of QoL had the most significant effect size (η^2^=0.14), in addition to our primary result, perceived stress. We attribute this improvement to the helper system that asked participants to find a helper, someone who can talk to and confide in about work. Participants also provided feedback that using this app with a helper allowed them to be more engaged in using the program and made the work environment more enjoyable. Improved social relationships within the workplace are estimated to increase employee well-being and company efficiency [48], constituting one reason for including CBT.

### Changes in Depression and Anxiety Levels and Other Characteristics of the Program

Although our initial analysis showed that the BetterLife program significantly improved BDI and BAI levels, this significance did not survive the Bonferroni correction. We assume that this is because of the baseline observation carried forward method used to create the missing data of the participants who dropped out, which is one of the most conservative ways to estimate missing data. Notably, the per-protocol analysis showed that BDI and BAI scores were significantly improved using the BetterLife program with moderate effect size (*F*=10.15, *P*=.002, η^2^=0.08 and *F*=8.21, *P*=.0049, η^2^=0.07, respectively). The program incorporated modules that directly help deal with depression and anxiety, as controlling psychological symptoms is essential in reducing perceived stress. The efficacy of web-based CBTs has been proven in patients with major depressive disorder and anxiety disorder with mild and moderate symptoms [13]. Although the current intervention was aimed at employees with work stress, future trials could be performed on clinical groups to explore the program’s effects.

The dropout rate of the intervention was 14.3%, slightly lower than the average dropout rate of internet-based CBTs according to a recent meta-analysis [49]. This, along with improved stress reduction, work engagement, and QoL, could result from the way the program was tailored for each participant, as described in the intervention section. The use of a helper combined with personalization as the user was guided through the program is a unique feature that makes BetterLife different from other internet-based programs dealing with stress. The feedback from the tests that participants took during the trial might also be a reason for the low degree of dropouts.

Although our study design did not include a direct comparison with face-to-face CBT, recent research comparing internet-based and face-to-face CBT showed similar results. Peter et al [14] showed similar efficacy in treating insomnia when comparing an online CBT with face-to-face CBT. A recent randomized noninferiority clinical trial showed online CBT to be noninferior to face-to-face CBT for health anxiety [50]. A systemic review and meta-analysis comparing online CBT and face-to-face CBT in psychiatric and somatic disorders reported that most disorders showed equivalent effects between the two groups [49]. These results emphasize the opinion that online CBT has become a viable and cost-effective option for face-to-face CBT, possibly because of advancements in technology and our familiarity with it. Unfortunately, we could not find any studies comparing online CBT with face-to-face CBT for stress reduction. Future studies are needed for stress management in this context.

### Limitations

Our study has several limitations. First, only psychological scales were used in the trial. Although using objective measurements such as heart rate variance or blood cortisol levels would have complemented the results, we avoided this so that the participants would not have to visit a psychiatric clinic. Stigma regarding psychiatric interventions remains widespread in Korea, and having participants with work stress visit a psychiatric ward could negatively impact the trial [51]. Second, the average perceived stress was relatively high in both groups. Generalizing this result to a population with low perceived stress would be difficult with the current data. Third, most participants were female, and most had an education level of college diploma or higher. Although there are similar findings regarding demographics [12,46], this should be accounted for when applying the results to a broader population. Finally, only pre- and postintervention data were collected. Additional data from months after the intervention are needed to examine if the effects of the intervention persist over time.

### Conclusions

BetterLife, a smartphone-based individually tailored CBT, effectively reduced stress and increased work engagement and QoL in people with work-related stress. This is a viable option for reducing workplace stress.

## Appendix

Multimedia Appendix 1 Screenshot of the BetterLife app.

Multimedia Appendix 2 Videorecording of a smartphone screen using the BetterLife app.

Multimedia Appendix 3 Result of paired *t* test of baseline and follow-up of the control group.

Multimedia Appendix 4 Result of split-plot analysis of variance for stress-related factors (unadjusted).

Multimedia Appendix 5 Result of per-protocol analysis of split-plot analysis of covariance for stress-related factors.

Multimedia Appendix 6 Result of per-protocol analysis of split-plot analysis of variance for additional outcome.

Multimedia Appendix 7 Dropout analysis of demographic characteristics of participants.

Multimedia Appendix 8 Dropout analysis of baseline work stress-related information.

Multimedia Appendix 9 CONSORT-eHEALTH checklist (V 1.6.1).

## Abbreviations

ANCOVA: analysis of covariance
ANOVA: analysis of variance
BAI: Beck Anxiety Inventory
BDI: Beck Depression Inventory–II
CBT: cognitive behavioral therapy
GAD-7: General Anxiety Test–7
MDI: Major Depression Inventory
PSS: Perceived Stress Scale–10
QoL: quality of life
RCT: randomized controlled trial
UWES: Korean Utrecht work engagement scale–9
WHO-5: World Health Organization Well-Being Index
WHOQOL: World Health Organization quality of life assessment, abbreviated
