# Supplementary figures and images for: The Efficacy of a Smartphone-Based App on Stress Reduction: Randomized Controlled Trial
Source: J Med Internet Res. 2022 Feb 15;24(2):e28703. doi: 10.2196/28703 (PMC8889477; doi:10.2196/28703)

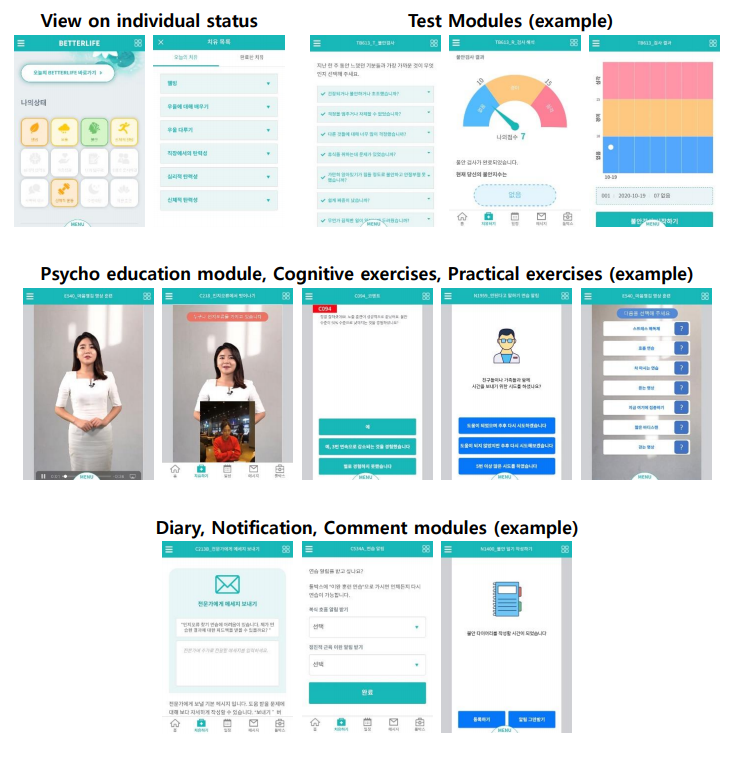

Supplement: Multimedia Appendix 1 [file jmir_v24i2e28703_app1.png]
