# Supplementary material for: The Efficacy of a Smartphone-Based App on Stress Reduction: Randomized Controlled Trial
Source: J Med Internet Res. 2022 Feb 15;24(2):e28703. doi: 10.2196/28703 (PMC8889477; doi:10.2196/28703)
Supplement: Multimedia Appendix 3 [file jmir_v24i2e28703_app3.docx]

Multimedia appendix 3. Result of paired t-test of baseline and follow up of the control group. ^a-b^

|  | | | Control (n=63) | | Test statistics | *P* |
| --- | --- | --- | --- | --- | --- | --- |
|  |  |  | Baseline | Follow up |  |  |
|  |  |  |  |  |  |  |
|  | PSS | | 20.1±3.8 | 19.6±4.4 | t=0.95 | .35 |
|  | UWESK Total | | 2.8±0.8 | 2.9±0.7 | t=-0.94 | .35 |
|  | WHOQOL | |  |  |  |  |
|  |  | Overall QoL | 3.1±0.7 | 3.3±0.8 | t=-1.15 | .25 |
|  |  | Overall health | 2.9±0.8 | 3.0±0.9 | t=-1.47 | .15 |
|  |  | Physical health | 58.4±12.3 | 60.7±11.8 | t=-1.54 | .13 |
|  |  | Psychological | 57.3±12.9 | 59.5±12.3 | t=-1.50 | .14 |
|  |  | Social relationship | 61.0±15.8 | 59.1±16.3 | t=1.01 | .31 |
|  |  | Environmental | 60.4±12.6 | 61.9±10.9 | t=-1.01 | .32 |
|  | Number of late days in past month | | 0.8±1.9 | 0.7±1.7 | t=0.89 | .38 |
|  | Number of early leave days in past month | | 0.3±0.9 | 0.3±0.9 | t=0.89 | .38 |
|  | Number of absent days in past month | | <0.1±0.1 | <0.1±0.3 | t=-0.44 | .66 |
|  | BDI | | 15.3±7.7 | 13.1±7.6 | t=2.80 | .007 |
|  | BAI | | 11.0±7.3 | 9.0±7.0 | t=2.24 | .03 |

^a^p<0.0038 was perceived to be significant

^b^PSS= Perceived Stress Scale; UWES= Utrecht Work Engagement Scale; WHOQOL= World Health Organization Quality of Life Scale, abbreviated; BDI= Beck Depression Inventory; BAI= Beck Anxiety Inventory
