# Supplementary material for: The Efficacy of a Smartphone-Based App on Stress Reduction: Randomized Controlled Trial
Source: J Med Internet Res. 2022 Feb 15;24(2):e28703. doi: 10.2196/28703 (PMC8889477; doi:10.2196/28703)
Supplement: Multimedia Appendix 4 [file jmir_v24i2e28703_app4.docx]

Multimedia appendix 4. Result of split-plot ANOVA for stress-related factors (unadjusted). ^a-c^

|  | | | Intervention (n=63) | | Control (n=63) | | Test statistics | *P* |
| --- | --- | --- | --- | --- | --- | --- | --- | --- |
|  | | | Baseline | Follow up | Baseline | Follow up |  |  |
|  |  |  |  | |  | |  |  |
|  | PSS | | 21.6±5.9 | 15.4±4.7 | 20.1±3.8 | 19.6±4.4 | F=27.31, η^2^=0.18 | <.001 |
|  | UWESK Total | | 2.6±0.8 | 3.1±0.9 | 2.8±0.8 | 2.9±0.7 | F=10.01, η^2^=0.08 | .002 |
|  | WHOQOL | |  |  |  |  |  |  |
|  |  | Overall QoL | 3.0±0.8 | 3.6±0.7 | 3.1±0.7 | 3.3±0.8 | F=9.72, η^2^=0.07 | .002 |
|  |  | Overall health | 2.9±0.9 | 3.3±0.8 | 2.9±0.8 | 3.0±0.9 | F=2.67 | .11 |
|  |  | Physical health | 56.1±13.8 | 65.8±14.1 | 58.4±12.3 | 60.7±11.8 | F=10.60, η^2^=0.08 | .001 |
|  |  | Psychological | 52.5±15.1 | 63.6±16.7 | 57.3±12.9 | 59.5±12.3 | F=15.40, η^2^=0.11 | <.001 |
|  |  | Social relationship | 55.1±18.1 | 66.6±14.6 | 61.0±15.8 | 59.1±16.3 | F= 21.43, , η^2^=0.15 | <.001 |
|  |  | Environmental | 58.2±14.6 | 68.6±12.5 | 60.4±12.6 | 61.9±10.9 | F= 12.24, η^2^=0.09 | .001 |
|  | BDI | | 17.7±9.5 | 11.5±9.2 | 15.3±7.7 | 13.1±7.6 | F=9.67, η^2^=0.07 | .002 |
|  | BAI | | 13.8±9.3 | 8.0±8.7 | 11.0±7.3 | 9.0±7.0 | F=8.02, η^2^=0.06 | .0054 |

^a^Statistics reported are for interaction between intervention and time of each variable. F(1,124).

^b^p<0.005 was perceived to be significant

^c^PSS = Perceived Stress Scale; UWES = Utrecht Work Engagement Scale; WHOQOL= World Health Organization Quality of Life Scale, abbreviated; BDI = Beck Depression Inventory; BAI = Beck Anxiety Inventory
