# Supplementary material for: The Efficacy of a Smartphone-Based App on Stress Reduction: Randomized Controlled Trial
Source: J Med Internet Res. 2022 Feb 15;24(2):e28703. doi: 10.2196/28703 (PMC8889477; doi:10.2196/28703)
Supplement: Multimedia Appendix 5 [file jmir_v24i2e28703_app5.docx]

Multimedia appendix 5. Result of per protocol analysis of split-plot ANCOVA for stress-related factors. ^a-c^

|  | | | Intervention (n=54) | | Control (n=61) | | Test statistics | *P* |
| --- | --- | --- | --- | --- | --- | --- | --- | --- |
|  | | | Baseline | Follow up | Baseline | Follow up |  |  |
|  |  |  |  | |  | |  |  |
|  | PSS | | 22.7±5.6 | 15.4±5.0 | 20.2±3.9 | 19.6±4.5 | F=30.89, η^2^=0.22 | <.001 |
|  | UWESK Total | | 2.6±0.8 | 3.2±0.9 | 2.9±0.7 | 2.9±0.7 | F=10.20, η^2^=0.08 | .002 |
|  | WHOQOL | |  |  |  |  |  |  |
|  |  | Overall QoL | 2.9±0.8 | 3.6±07 | 3.2±0.7 | 3.3±0.8 | F=10.20, η^2^=0.08 | .002 |
|  |  | Overall health | 2.9±0.9 | 3.4±0.8 | 2.9±0.9 | 3.0±0.9 | F= 2.33 | .13 |
|  |  | Physical health | 56.0±13.7 | 67.4±13.4 | 58.2±12.4 | 60.6±11.9 | F= 11.41, η^2^=0.09 | .001 |
|  |  | Psychological | 52.4±15.4 | 65.4±16.6 | 57.4±13.1 | 59.7±12.5 | F= 17.12, η^2^=0.13 | <.001 |
|  |  | Social relationship | 52.6±17.9 | 66.0±15.1 | 60.9±16.0 | 59.0±16.5 | F= 22.15, η^2^ =0.17 | <.001 |
|  |  | Environmental | 57.9±15.4 | 70.1±12.4 | 60.6±12.8 | 62.2±11.0 | F= 12.51, η^2^=0.10 | <.001 |
|  | BDI | | 18.0±9.9 | 10.9±9.4 | 15.3±7.8 | 13.1±7.7 | F=10.15, η^2^=0.08 | .002 |
|  | BAI | | 14.2±9.5 | 7.4±8.7 | 10.9±7.4 | 8.9±7.0 | F=8.21, η^2^=0.07 | .0049 |

^a^Statistics reported are for interaction between intervention and time of each variable. F(1,112).

^b^p<0.005 was perceived to be significant

^c^PSS = Perceived Stress Scale; UWES = Utrecht Work Engagement Scale; WHOQOL= World Health Organization Quality of Life Scale, abbreviated; BDI = Beck Depression Inventory; BAI = Beck Anxiety Inventory
