# Supplementary material for: The Efficacy of a Smartphone-Based App on Stress Reduction: Randomized Controlled Trial
Source: J Med Internet Res. 2022 Feb 15;24(2):e28703. doi: 10.2196/28703 (PMC8889477; doi:10.2196/28703)
Supplement: Multimedia Appendix 6 [file jmir_v24i2e28703_app6.docx]

Multimedia appendix 6. Result of per protocol analysis of split-plot ANOVA for additional outcome. ^a, b^

|  | | | Intervention (n=54) | | Control (n=61) | | Test statistics | *P* |
| --- | --- | --- | --- | --- | --- | --- | --- | --- |
|  | | | Baseline | Follow up | Baseline | Follow up |  |  |
|  |  |  |  | |  | |  |  |
|  | Number of late days in past month | | 0.7±1.7 | 0.3±0.8 | 0.9±1.9 | 0.7±1.7 | F=0.65 | .42 |
|  | Number of early leave days in past month | | 0.3±0.6 | 0.1±0.5 | 0.3±1.0 | 0.3±0.9 | F=0.97 | .33 |
|  | Number of absent days in past month | | <0.1±0.2 | <0.1±0.1 | <0.1±0.1 | <0.1±0.3 | F=0.50 | .48 |

^a^Statistics reported are for interaction between intervention and time of each variable. F(1,113).

^b^p<0.05 was perceived to be significant
