# Supplementary material for: The Efficacy of a Smartphone-Based App on Stress Reduction: Randomized Controlled Trial
Source: J Med Internet Res. 2022 Feb 15;24(2):e28703. doi: 10.2196/28703 (PMC8889477; doi:10.2196/28703)
Supplement: Multimedia Appendix 7 [file jmir_v24i2e28703_app7.docx]

Multimedia appendix 7. Dropout analysis of demographic characteristics of participants. ^a, b^

|  | | | Dropout (n=11) | Per protocol (n=115) | Test statistics | *P* |
| --- | --- | --- | --- | --- | --- | --- |
|  |  |  |  |  |  |  |
| **General demographic information** | | | | | |  |
|  | Age (years, mean ± standard deviation) | | 41.8±9.6 | 37.6±9.2 | t=-1.45 | .15 |
|  | Gender (male (%) / female (%)) | | 2(18.2%)/9(81.8%) | 23(20.0%)/92(80.0%) | χ^2^=0.02 | .89 |
|  | Education | |  |  | FET=3.12 | .18 |
|  |  | High school | 4 (36.4%) | 18 (15.7%) |  |  |
|  |  | Undergraduate | 7 (63.6%) | 84 (73.0%) |  |  |
|  |  | Graduate | 0 (0.0%) | 13 (11.3%) |  |  |
|  | Marital state (single/married) | | 5(45.5%)/6(54.5%) | 54(47.0%)/61(53.0%) | χ^2^=0.01 | .92 |
|  | Alcohol (yes/no) | | 10(90.9%)/1(9.1%) | 93(80.9%)/22(19.1%) | FET | .69 |
|  | Smoking (yes/no) | | 0(0.0%)/11(100.0%) | 11(9.6%)/104(90.4%) | FET | .60 |
| **Workplace information** | | | | | |  |
|  | Company size (employee) | |  |  | FET=4.86 | .22 |
|  |  | Under 10 | 2 (18.2%) | 8 (7.0%) |  |  |
|  |  | 10~29 | 0 (0.0%) | 8 (7.0%) |  |  |
|  |  | 30~99 | 0 (0.0%) | 22 (19.1%) |  |  |
|  |  | 100~299 | 0 (0.0%) | 9 (7.8%) |  |  |
|  |  | Over 300 | 9 (81.8%) | 68 (59.1%) |  |  |
|  | Types of work fields | |  |  | FET=6.92 | .16 |
|  |  | Sales, Services | 1 (9.1%) | 3 (2.6%) |  |  |
|  |  | Technical | 0 (0.0%) | 5 (4.3%) |  |  |
|  |  | Office | 1 (9.1%) | 36 (31.3%) |  |  |
|  |  | Professional | 4 (36.4%) | 41 (35.7%) |  |  |
|  |  | Civil servant/Teacher | 0 (0.0%) | 9 (7.8%) |  |  |
|  |  | Others^c^ | 5 (45.5%) | 21 (18.3%) |  |  |
|  | Type of employment | |  |  | χ^2^=0.42 | .52 |
|  |  | Regular position | 10 (90.9%) | 96 (83.5%) |  |  |
|  |  | Temporary position | 1 (9.1%) | 19 (16.5%) |  |  |
|  | Job grade | |  |  | FET =4.31 | .48 |
|  |  | Staff | 0 (0.0%) | 3 (2.6%) |  |  |
|  |  | Administrative manager | 0 (0.0%) | 24 (20.9%) |  |  |
|  |  | Assistant manager | 2 (18.2%) | 22(19.1%) |  |  |
|  |  | General manager | 4 (36.4%) | 25 (21.7%) |  |  |
|  |  | Director and higher | 4 (36.4%) | 27 (23.5%) |  |  |
|  |  | No job grade | 1 (9.1%) | 14 (12.2%) |  |  |
|  | Customer complaints (yes/no) | | 6(54.5%)/5(45.5%) | 81(70.4%)/34(29.6%) | χ^2^=1.19 | .28 |
|  | Income | |  |  | FET=4.26 | .31 |
|  |  | Under 2 million won | 5 (45.5%) | 20 (17.4%) |  |  |
|  |  | 2–3 million won | 5 (45.5%) | 59 (51.3%) |  |  |
|  |  | 3–4 million won | 1 (9.1%) | 16 (13.9%) |  |  |
|  |  | 4–5 million won | 0 (0.0%) | 13 (11.3%) |  |  |
|  |  | Over 5 million won | 0 (0.0%) | 7 (6.1%) |  |  |
|  | Work days/week ^d^ | | 5.0±0.0 | 5.04±0.3 | t=0.44 | .66 |
|  | Work hours/day ^d^ | | 8.0±0.0 | 8.3±0.8 | t=3.63 | <.001 |
|  | Work experience in current work field (months) | | 125.2±94.0 | 131.1±98.2 | t=0.19 | .85 |
|  | Work experience in current job (months) | | 86.0±87.2 | 100.3±98.2 | t=0.47 | .64 |

^a^FET= Fisher’s Exact Test

^b^p<0.05 was perceived to be significant

^c^Others include miscellaneous position in company under 9 employees, management position of a company with over 10 employees, etc.

^d^one value missing
