# Supplementary material for: The Efficacy of a Smartphone-Based App on Stress Reduction: Randomized Controlled Trial
Source: J Med Internet Res. 2022 Feb 15;24(2):e28703. doi: 10.2196/28703 (PMC8889477; doi:10.2196/28703)
Supplement: Multimedia Appendix 8 [file jmir_v24i2e28703_app8.docx]

Multimedia appendix 8. Dropout analysis of baseline work stress-related information. ^a, b^

|  | | | Dropout (n=11) | | Per protocol (n=115) | *P* |
| --- | --- | --- | --- | --- | --- | --- |
|  |  |  |  |  |  |  |
|  | PSS | | 15.9±2.9 | 21.4±4.9 | t=3.63 | <.001 |
|  | UWES | | 2.7±0.8 | 2.8±0.8 | t=0.31 | .76 |
|  | WHOQOL | |  |  |  |  |
|  |  | Overall QoL | 3.3±0.6 | 3.0±0.8 | t=-0.93 | .35 |
|  |  | Overall health | 2.8±0.6 | 2.9±0.9 | t=0.22 | .83 |
|  |  | Physical health | 57.4±14.2 | 57.2±13.0 | t=-0.04 | .97 |
|  |  | Psychological | 52.8±12.3 | 55.1±14.4 | t=0.50 | .62 |
|  |  | Social relationship | 68.7±10.5 | 57.0±17.4 | t=-2.19 | .03 |
|  |  | Environmental | 58.6±8.6 | 59.4±14.0 | t=0.17 | .87 |
|  | BDI | | 15.1±6.2 | 16.6±8.9 | t=0.55 | .58 |
|  | BAI | | 12.2±7.3 | 12.4±8.5 | t=0.10 | .92 |
|  | Number of late days in past month | | 0.8±1.8 | 0.8±1.8 | t=-0.08 | .94 |
|  | Number of early leave days in past month | | 0.0±0.0 | 0.3±0.8 | t=4.25 | <.001 |
|  | Number of absent days in past month | | 0.1±0.3 | 0.0±0.2 | t=-0.70 | .50 |

^a^p<0.05 was perceived to be significant

^b^PSS= Perceived Stress Scale; UWES= Utrecht Work Engagement Scale; WHOQOL= World Health Organization Quality of Life Scale, abbreviated; BDI= Beck Depression Inventory; BAI= Beck Anxiety Inventory
